# Insulin resistance/hyperinsulinemia: an important cardiovascular risk factor that has long been underestimated

**DOI:** 10.3389/fcvm.2024.1380506

**Published:** 2024-03-13

**Authors:** Serafino Fazio, Valentina Mercurio, Loredana Tibullo, Valeria Fazio, Flora Affuso

**Affiliations:** ^1^ Department of Internal Medicine, Federico II University Hospital, Naples, Italy; ^2^ UOC.Medicina Interna, Azienda Ospedaliera di Caserta, Caserta, Campania, Italy; ^3^ Independent Researcher, Naples, Campania, Italy

**Keywords:** insulin signaling, insulin resistance, hyperinsulinemia, cardiovascular risk factors, cardiovascular diseases

## Abstract

Cardiovascular mortality is still excessively high, despite the considerable progress made in the prevention and treatment of cardiovascular diseases. Although many cardiovascular risk factors (such as arterial hypertension, hypercholesterolemia, diabetes, etc.), identified in the general population, are being promptly treated, to date little consideration is given to a cardiovascular risk factor which we believe has largely demonstrated in the scientific literature of the last three decades that, if neglected, can produce a series of relevant negative effects on the cardiovascular system: insulin resistance (IR)/hyperinsulinemia (Hyperins). This risk factor is still not sufficently sought in the general population and, consequently, is not treated promptly, as it should be, to avoid its negative impact on the cardiovascular system. IR's prevalence is constantly growing worldwide, and it is estimated to have reached a prevalence of 51% of the general population in developed and developing countries, and Hyperins is a constant and strong feature of IR. This article aims to stimulate the scientific community towards IR/Hyperins as relevant cardiovascular risk factor, since it is still neglected. The scientific literature analyzed and used to for this article was found on PubMed, Scopus, Science Direct, etc, using the following keywords: insulin, insulin signaling, insulin resistance, hyperinsulinemia, cardiovascular risk factors, cardiovascular system, cardiovascular diseases. We selected studies that explored the association between IR/Hyperins and the cardiovascular system, and those that discussed the possibilities of screening and treatment of IR/Hyperins.

## Introduction

It is clear to everyone that, despite the significant progress made in the prevention and treatment of cardiovascular diseases, cardiovascular mortality is still excessively high. It is estimated that every year in Europe there are 2 million deaths from cardiovascular causes (1), and deaths from cardiovascular causes are still in first place among the various causes of death. Indeed, there is a cardiovascular risk factor, which is pathophysiologically connected with the other well-recognized cardiovascular risk factors, treated according to the latest guidelines, which is still not fully taken into consideration: insulin resistance/hyperinsulinemia (IR/Hyperins). This important cardiovascular risk factor is not screened in the general population and, consequently, is not treated, as it is done, instead, with the other recognized cardiovascular risk factors.

It is estimated that IR's prevalence is constantly growing worldwide, reaching up to 51% of the general population, with the highest values for developed and/or developing countries (2). Hyperins is a constant and prominent feature of IR (3, 4). Insulin is a hormone that, in addition to the regulation of glucose metabolism, has important actions in several systems and organs, with the cardiovascular system as one of its main targets, and like all hormones it can cause damage if its levels are outside the normal range (5). Unfortunately, Hyperins that accompanies IR is mostly asymptomatic or paucisymptomatic, making its early diagnosis very difficult. Hence, Hyperins associated with IR lasts, in most cases, for many years before showing itself to have caused some damage or, more often, to have resulted in overt type 2 diabetes (3, 4). In this article we aim at analyzing the evidence in the scientific literature for considering IR/Hyperins as an important CV risk factor, like or more than other well-recognized CV risk factors, and verifying if there are possibilities of mass screening and, consequently, of timely treatment. The scientific literature consulted and used to write the article was found in PubMed, Scopus, Science Direct, etc. using the following keywords: insulin, insulin signaling, insulin resistance, hyperinsulinemia, cardiovascular risk factors, cardiovascular diseases, cardiovascular system. We selected the studies that explored the association between IR/Hyperins and the cardiovascular system, and those that discussed the possibilities of screening and treatment of IR/Hyperins.

## Insulin resistance/hyperinsulinemia

IR is characterized by a decrease in sensitivity of the body's cells to insulin. Consequently, the action of insulin in regulating blood sugar is reduced compared to normal and, as an effect of this alteration, a condition of compensatory Hyperins (the increase in circulating insulin levels) is generated: the cells do not internalize glucose because they do not respond normally to insulin and, thus, the pancreas secretes more insulin to maintain normal blood sugar levels (3, 4). Therefore, Hyperins is a constant and relevant feature of the IR. Over time (years), the pancreas will no longer succeed in its compensatory maneuver of increasing insulin secretion, therefore subjects with IR will degenerate towards prediabetes and, subsequently, towards overt type 2 diabetes (3, 4).

The causes of IR are various and not entirely understood: overweight and obesity (particularly visceral); sedentary lifestyle; unbalanced diet in favor of excessive carbohydrate intake; chronic stress; prolonged use of diabetogenic drugs; genetic causes; etc. Muscle tissue, adipose tissue, and the liver are the main targets of insulin resistance. The symptoms and signs of IR are scarce and non-specific (drowsiness and tiredness, increased appetite, concentration difficulties, tendency to gain weight, predominantly abdominal adiposity, high levels of LDL cholesterol, high fasting triglyceride levels, tendency to arterial hypertension, etc.) (3, 4, 6). For this reason, IR/Hyperins lasts, often unrecognized, for many years. Diagnosis is easier in subjects with metabolic syndrome and in infertile women with polycystic ovary syndrome, but IR can also be present in subjects that are difficult to suspect, such as in normal weight and thin subjects (7).

For this reason, this condition remains unrecognized for many years, being discovered only when prediabetes or overt diabetes appear, or when a cardiovascular complication occurs. At the basis of IR/Hyperins there may be a reduced number of insulin receptors or, more often, a defect in post-receptorial signal transduction of insulin receptor. The binding of insulin to its receptor in target tissues leads to the activation of complex pathways, which regulate the transcription of target genes. Two major signaling pathways have been identified: (A) The phosphoinositide-3 kinase-dependent pathway (PI3-K), which primarily mediates insulin metabolic pathways, including the regulation of glucose metabolism in muscle, adipose tissue, and liver, and the regulation of nitric oxide (NO) production by the endothelium and vascular smooth muscle cells (VSMC). (B) The mitogen-activated protein kinase (MAPK)-dependent pathway, which mediates the nonmetabolic, but equally important, actions of insulin, including its mitogenic and proliferative actions, and the secretion of endothelin-1 (ET-1) by of the endothelial cells.

Under IR conditions, these pathways, which contribute to normal metabolic and cardiovascular homeostasis, are unbalanced by a specifically prevalent alteration at the level of the PI3-K dependent metabolic pathway, so that compensatory Hyperins ends up overstimulating the MAPK dependent pathway. This produces severe cell growth and vasoconstriction due to the prevalence of ET-1 secretion over NO secretion, which is mediated by the altered PI3-K pathway, resulting in significant endothelial dysfunction, which, as it is well known, underlies the development and progression of the atherosclerosis (8–12).

Moreover, Hyperins produces hyperactivation of the sympathetic system and stimulates the reabsorption of sodium in the renal tubules, thus facilitating the onset of arterial hypertension (13, 14). All this, if not counteracted, can determine important structural and functional alterations of the cardiovascular system overtime. For this reason, it would be extremely important, as for the other cardiovascular risk factors, to carry out an early screening of the IR/Hyperins, in order to promptly intervene with a preventive treatment.

The gold standard for the diagnosis of IR is the euglycemic-hyperinsulinemic clamp, which, however, being complex and expensive, is used only for scientific research purposes. However, for screening purposes, two surrogate indices that have been shown to correlate with clamp could be used: 1. The Homeostatic Model Evaluation Index (HOMA-IR); 2. The triglyceride glucose index (TyG). The former is based on the simultaneous dosage of fasting insulin and blood glucose [HOMA-IR = (glucose mmol/L × insulin mU/L)/22.5], and its normal value in adults is between 0.23 and 2.5, while in children it reaches up to 3.6. TyG is calculated by first taking the logarithm of the product of triglycerides and fasting glucose, and then dividing by 2 [TyG = log (triglycerides × glucose)/2]. The normal value is less than 4.5 (15, 16).

## Effects of insulin resistance/hyperinsulinemia on cardiovascular system

**Figure 1:**
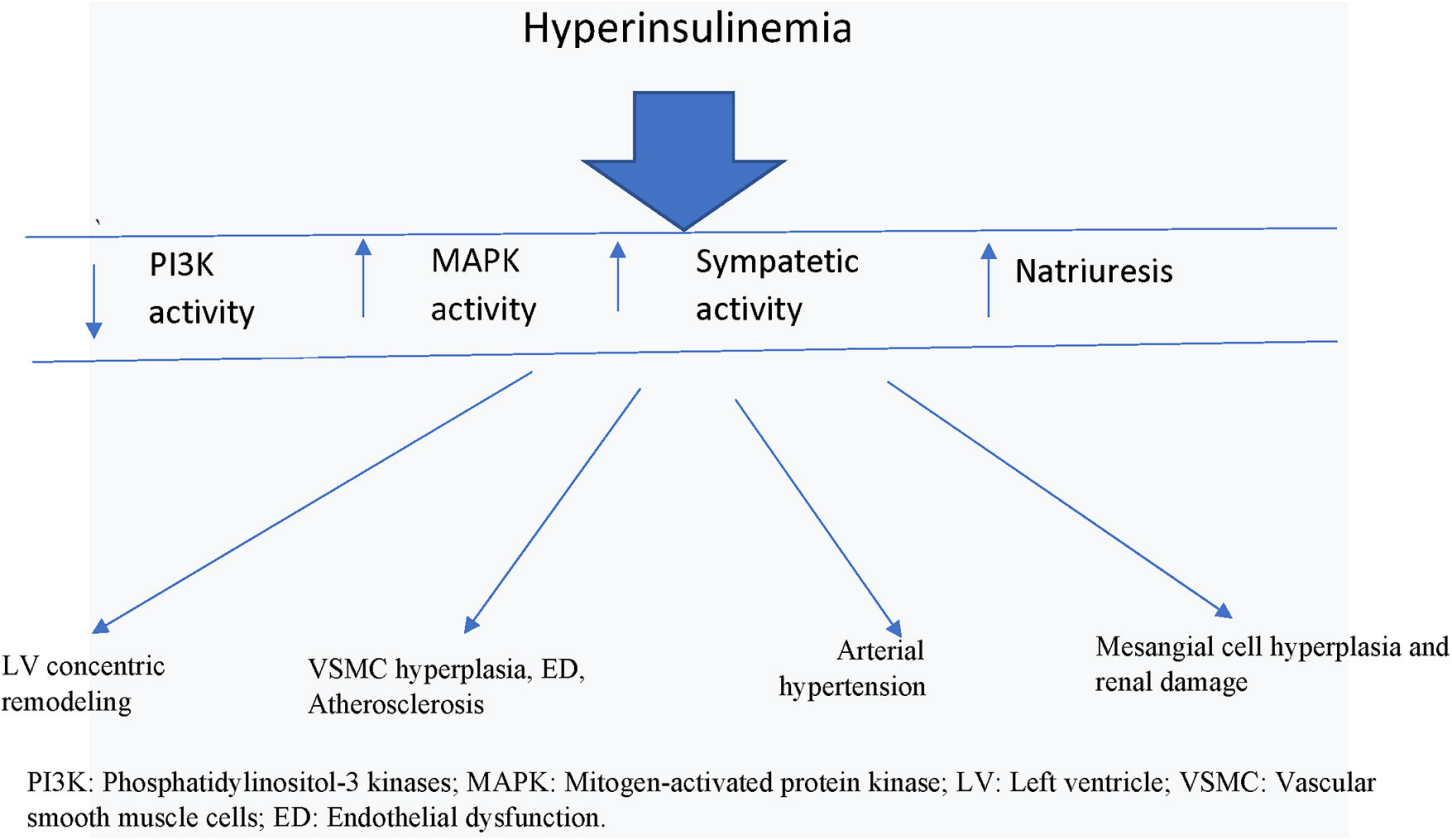
Actions of hyperinsulinemia on cardiovascular system. PI3K, Phosphatidylinositol-3 kinases; MAPK, mitogen-activated protein kinase; LV, left ventricle; VSMC, vascular smooth muscle cells; ED, endothelial dysfunction.

Globally, in recent decades, there have been significant improvements in mortality rates in all continents, even if critical situations remain in some poor countries. In the European Union (EU), in 2016, such rates were more comforting than the world average, and Italy was one of the most virtuous countries along with France and Spain. Cardiovascular diseases, tumors and respiratory diseases are the main causes of death in Italy and in Europe, cumulatively accounting for about 70% of total deaths (17). Despite the considerable progress made in the prevention and treatment, cardiovascular diseases are still the first cause of death in Europe with a percentage of 33% of total deaths in 2020 (18). For this reason, we must try to do more in the preventive field of cardiovascular diseases. In particular, we believe that the risk factor IR/Hyperins has been considered a marginal risk factor for too many years, while important preventive efforts are implemented only when this turns into overt diabetes. Unfortunately, it could be too late, because IR/Hyperins, preceding diabetes by many years (even up to 15 years) (4), most likely, could have already caused important cardiovascular alterations, so that, according to prevention guidelines, the diabetes is treated in secondary prevention, that is, as if a first cardiovascular event has already occurred in that patient. Indeed, when we carry out an initial instrumental verification in a patient with recent onset of type 2 diabetes, we easily realize that there are already evident cardiovascular alterations, such as left ventricular remodeling and atherosclerosis at various explorable vascular districts (19) (Figure 1).

There is a vast scientific literature supporting a negative action of IR/Hyperins on the cardiovascular system (20–25). On the other hand, insulin is a hormone, even a rather important one, and, like all the hormones that regulate our body functions, it will cause damage both if the circulating levels are low and if, as in the case of IR, they are high for many years. This is a rather simplistic interpretation of the thing, however, if we examine the problem more in detail, we find considerable scientific literature to support negative actions of IR/Hyperins on the CV system. It is well known that endothelial dysfunction (ED) appears early in the development of the atherosclerotic process. One of the main mechanisms through which IR/Hyperins determines ED is due to the malfunction of the PI3-K dependent signaling pathway. This produces an imbalance between the reduced production of NO, linked to the bad functioning of this pathway, and the increased secretion of ET-1 by the endothelial cells, linked to the signaling of the MAPK dependent pathway, which remains practically unchanged and subject to the action of greater quantities of insulin. This causes vasoconstriction with alteration of the district flows (26).

Furthermore, increased levels of circulating insulin via common insulin-like growth factor-1 (IGF-1) receptors and the MAPK-dependent signaling pathway stimulates VSMC and endothelial cell proliferation, resulting in increased wall thickness of the vessels, loss of vascular elasticity, and parietal calcifications, so that the process of atherosclerosis is triggered (27) (Figure 1).

Therefore, under IR/Hyperins conditions, the excessive stimulation of the MAPK cascade, which has vasoconstrictive (↥ ET-1) and growth-promoting effects at various cellular levels, ends up determining part of the deleterious effects at the cardiovascular level. At this point there is also solid evidence to support the fact that Hyperins may also be a determinant of arterial hypertension, in fact it has been demonstrated that insulin causes retention of sodium and water in the kidney, and that both endogenous and exogenous Hyperins were found related to increases in blood pressure (28) (Figure 1). In fact, insulin receptors have been detected in the renal tubules, and their stimulation by insulin has been shown to produce increased sodium reabsorption. In addition to the mechanisms described above, there is a clear and demonstrable relationship between insulin levels and sympathetic nervous system activity (Figure 1). Increased blood pressure has been shown to be associated with both insulin levels and sympathetic nervous system activity, a relationship that was noted both in the general population and after adjustment for indexed body mass and body fat distribution. Furthermore, discontinuation of Hyperins in obese subjects resulted in a reduction in plasma norepinephrine levels and blood pressure (14).

Sympathetic hyperactivation characterizes IR/Hyperins conditions and may contribute to the increased cardiovascular morbidity and, in turn, to the reduced insulin sensitivity of these patients (29) (Figure 1). During euglycemic clamp with two different increased doses of insulin it was verified that the increase in insulin levels produced significant increases in circulating norepinephrine levels (from 119 ± 19 pg/ml to 258 ± 25 *p* < 0.02 and 285 ± 95 *p* < 0.01, respectively) (30, 31).

As already mentioned, insulin is also a growth factor and, as such, the conditions of IR/Hyperins have been found to be associated with an increased prevalence of chronic kidney disease, and there are numerous studies supporting the existence of numerous mechanisms that link IR/Hyperins with kidney damage. Insulin, by itself, promotes the proliferation of renal mesangial cells and stimulates the production of other important growth factors such as insulin-like growth factor-1 and transforming growth factor beta. Insulin also upregulates the expression of angiotensin II type 1 receptor in the mesangial cells, thereby increasing the damaging effects of angiotensin II in the kidney, and stimulates the production and action of ET-1 in the kidneys, while reducing the renal vascular production of NO. These mechanisms are implicated in the development and progression of diabetic nephropathy (32).

The actions of insulin on the mass and remodeling of the left ventricle are also extremely important. Indeed, it is known that left ventricular mass (LVM) is associated with an increased risk of cardiovascular events (33). Furthermore, increased left ventricular mass, documented by electrocardiography or echocardiography, has been shown to be an independent risk factor for the development of heart failure (HF) (34). An additional study also found that LVM index is an independent predictor of sudden cardiac death, and may help to improve sudden death risk prediction along with other conventional cardiovascular risk factors (35) (Figure 1).

A recent study demonstrated, in 88 black sub-Saharan African hypertensive patients with left ventricular hypertrophy (LVH), that obesity and IR/Hyperins were the primary predictors of LVH, and suggested that, among various risk factors, particular emphasis had to be placed on the correction of obesity and IR (36). In another study carried out in Japan it was shown, by echocardiography in 210 normotensive subjects and 180 mildly or moderately hypertensive subjects, that the sum of glucose levels (or hemoglobin A1c levels) in all subjects, and the sum of insulin levels (or 2-hour post-load glucose insulin), in subjects without diabetes mellitus, correlated significantly with LV relative wall thickness (RWT) independent of age, systolic blood pressure, and body mass index. The authors concluded that hyperglycemia and Hyperins could promote concentric LV changes in normotensive and mildly/moderately hypertensive subjects (37). Other investigators have demonstrated that IR/Hyperins and waist-to-hip ratio are closely associated with concentric LV remodeling, independent of BMI, and suggest that IR/Hyperins treatment could give an important boost to regression of the concentric remodeling of the LV (38). In studies of our group we verified, in 59 pts with metabolic syndrome and IR/Hyperins, the presence of increased LVMI and RWT associated with LV diastolic dysfunction (39, 40).

IR/Hyperins has also been shown to be highly prevalent (67%) in non-diabetic patients with chronic heart failure (HF). In these patients it has been verified that the fasting IR indices progressively increase with the worsening of the New York Heart Association class. Furthermore, patients with HF and IR had significantly lower exercise capacity and peak O2 achieved than patients with HF but no evidence of IR (41). It is well known that LVH is a constant feature of diabetic cardiomyopathy, and is thought to be a strong predictor of adverse cardiovascular events, in particular, of HF with preserved ejection fraction (HFpEF). In fact, LVH can determine an alteration of the filling function of the LV and, consequently, diastolic HF (42). For this reason, it was hypothesized that the treatment of IR/Hyperins in patients with HF could lead to a slowing down of the negative evolution of HF.

In addition, it has been shown that inflammation may be the common pathway between insulin resistance, hypertension and other cardiovascular risk factors. Inflammation is involved in both hyperinsulinemia, hypertension and other cardiovascular conditions (43–45). In conditions of IR there is the production of pro inflammatory cytokines which determine a state of IR in the target tissues of insulin's actions (46, 47). An underlying inflammatory state that can cause IR/Hyperins, which in turn can cause inflammation creating a self-regenerating short circuit. Many studies have highlighted how chronic inflammation through the production of cytokines can be the basis of atherosclerosis and its progression (48, 49).

## Treatment of IR/hyperins

The first thing to do to improve the condition of IR/Hyperins is a radical change in lifestyle by increasing physical activity and reducing daily caloric intake through a balanced diet not rich in carbohydrates. However, clinical practice shows that only a low percentage of subjects consistently implement these lifestyle changes. For this reason, the majority of the insulin resistant population needs to be helped by adding to their diet substances that increase insulin sensitivity and reduce circulating levels of insulin. There are numerous substances, both drugs and natural substances, that act positively in this sense, but, even today, no substance is authorized for this purpose outside of the diagnosis of diabetes. Among these substances, certainly, Sodium-Glucose cotrasporter2-inhibitors (SGLT2-i), Metformin, Berberine, Glucagon Peptide -1 receptor agonists (GLP-1 Ras) and L-arginine deserve particular attention.

### Sodium-glucose cotransporter2-inhibitors

Sodium-glucose cotransporter2 inhibitors (SGLT2-i) are a relatively recent class of oral drugs, approved for the treatment of adults with type 2 diabetes and, recently, also for the treatment of HF (50, 51). They include canagliflozin, dapagliflozin, and empagliflozin. A vast scientific literature is now available demonstrating how treatment with SGLT2-i reduces the risk of hospitalization, death from cardiovascular events and all causes of death in patients with HF (52). A recent study on 6,263 patients >40 years old with EF > 40%, assigned to receive dapagliflozin (10 mg once daily) or placebo in addition to standard therapy, demonstrated a significant reduction in the risk of worsening of HF and death from cardiovascular events (53). Another recent study of metaregression-analysis of vaste proportion, aimed at assessing the effects of these drugs on allcause mortality, has shown that SGLT2-i reduce all-cause mortality in randomized trials (54).

The exact mechanisms by which these drugs determine their beneficial effects are not exactly known. However, the most likely hypothesized mechanisms are: regulation of blood volume, cardiorenal mechanisms, direct effects on contractility and sodium homeostasis, metabolic effects, improved cardiac remodeling, reduction of inflammation and oxidative stress, etc. (55). Administration of SGLT2-i to subjects with IR/Hyperins reduces the amount of blood glucose, due to the increased excretion of urinary glucose. For this reason, the amount of insulin required is reduced and, as consequence, the daily average levels of circulating insulin will be reduced compared to pretreatment (56). SGLT2-i reduce IR and insulin levels, consequently reducing their deleterious cardiovascular effects.

### Metformin

Metformin is a long-standing oral antidiabetic, belonging to biguanide class of drugs, which has many scientific demonstrations of effectiveness in reducing IR/Hyperins (57). A literature review study investigated the effects of Metformin on all-cause mortality and the incidence of ageing diseases in diabetic subjects compared to a non-diabetic population, and diabetic subjects treated with therapies different from Metformin. The results of the study highlighted that mortality was significantly reduced in diabetic subjects treated with Metformin, both compared to non-diabetics and diabetics not treated with Metformin. Furthermore, the group of patients treated with Metformin also had a reduction in cancer, compared to non-diabetics, and in cardiovascular diseases, compared to diabetics not taking Metformin (58).

Metformin treatment has also demonstrated notable benefits in patients with HFpEF. In fact, the results of a fairly recent study, carried out using meta-regression analysis on observational and randomized studies, shows that treatment with Metformin significantly reduces (*p* < 0.003) mortality in patients with HFpEF (59). Metformin has been shown to have numerous favorable effects on the heart of subjects with HF. In fact, it improves the energy status of the myocardium as a consequence of the modulation of glucose and lipid metabolism, the reduction of oxidative stress and inflammation, and the reduction of pathological cardiac remodeling (60). A few years ago the results of a meta-analysis were published demonstrating how metformin therapy has favorable effects on left ventricular mass (LVM) and EF both in subjects with and without preexisting cardiovascular disease (61).

### Berberine

Berberine is a plant alkaloid that has been in use for over 2000 years in Chinese and Indian Ayurvedic medicine, and has proven over time to have many beneficial effects on human health. It is contained in many plants including Coptis Chinensis. There is a large amount of scientific literature that supports the fact that berberine has favorable effects on cardiovascular risk factors such as hypercholesterolemia, hyperglycemia, arterial hypertension and insulin resistance (62–65).

Berberine, in addition to having many experimental studies to support its positive action on glucose metabolism and IR/Hyperins, also has many clinical studies that support its role in cardiovascular prevention. A review and meta-analysis of randomized controlled trials on berberine has highlighted how it can be a valid alternative, without causing serious adverse reactions, to drugs currently used for cardiovascular prevention (66). In a randomized, placebo -controlled study lasting 4 months, 59 patients with metabolic syndrome were treated with a combination of nutraceuticals, containing berberine 500 mg, or placebo. The results of this study demonstrated that pts treated with berberine had a significant reduction in IR/Hyperins, as documented by reductions in HOMA-IR and fasting insulin levels (39). Furthermore, in these pts, using Doppler-ecocardiography, was documented a significant reduction in LVM and relative wall thickness (RWT), and an improvement in LV diastolic function (40). Another randomized and controlled study of berberine 500 mg per day compared to placebo, carried out in 145 subjects with metabolic syndrome and left ventricular hypertrophy, demonstrated how treatment with berberine for 6 months resulted in a significant reduction (*p* < 0.001) in LVM. For this reason, the authors conclude that this treatment could represent an effective strategy to reduce an important risk factor such as LVM (67).

Also important is that, in a study carried out in 2 groups of pts with HF, 79 pts treated with berberine in addition to standard therapy and 76 pts treated with standard therapy plus placebo, during a 2-year follow-up, 7 pts died in the berberine group and 13 pts in the placebo group (*p* < 0.02) (68).

### Glucagon-like peptide 1 receptor agonists

Glucagon-like peptide 1 receptor agonists (GLP-1 RAs) are a class of drugs not long ago authorized for the treatment of type 2 diabetes and obesity. They are administered by subcutaneous injection and are quite expensive drugs. This class of drugs includes semaglutide, albiglutide, dulaglutide, exenatide, liraglutide, and lixisenatide. It seems that they act not only by reducing body weight, but also by acting on mechanisms involved in the determinism of IR/Hyperins, such as, for example, increasing the expression of glucose transporters in insulin-dependent tissues, reducing inflammation and oxidative stress, and modulating lipid metabolism (69, 70).

However, although GLP-1 RAs have actually been shown to improve cardiovascular risk factors such as body weight, blood pressure, LDL cholesterol and triglycerides, and glycemic control, they have been shown to reduce all-cause mortality in patients with type 2 diabetes at high risk of cardiovascular events, but have not reduced cardiovascular mortality, non-fatal myocardial infarction, non-fatal stroke and hospitalizations for HF (71, 72). Another recent meta-analysis carried out on 54,092 pts from 7 randomized placebo-controlled trials on the use of GLP-1 RAs in subjects with type 2 diabetes, of whom 16% also had a history of HF, demonstrated that these drugs appear to protect the diabetic population from the development of HF, but, in subjects with pre-existing HF, they do not reduce the onset of episodes of HF exacerbation with consequent hospitalization, nor mortality (73). A further recent metaanalysis carried out to verify whether treatment with GLP-1 RAs in subjects with HF, with or without type 2 diabetes, could lead to a reduction in morbidity and mortality compared to placebo, also demonstrated that this therapy did not reduce the number of major adverse cardiovascular events, including cardiovascular mortality or reduction in hospital admissions for HF, and did not lead to an improvement in HF or six-minute walking test (74).

In addition, recently, numerous reports of serious adverse events have been published, such as cases of severe pancreatitis. In fact, patients taking either semaglutide or liraglutide had nine times an elevated risk for pancreatitis but, also, they had a very high risk to develop bowel obstruction, and to experience gastroparesis (75). Furthermore, European Medicine Agency published a statement on ongoing review of GLP-1 RAs about the possibility that liraglutide and semaglutide can stimulate suicidal thoughts and self-injury, made by the Icelandic medicines agency (76).

### L-arginine

L-arginine is an amino acid defined as conditionally essential. This is because it is essential for growing subjects and malnourished adults suffering from serious illnesses or who have suffered major trauma or burns, but not for healthy adult subjects, because it can be synthesized by the body and is regularly taken in with the diet in a quantity that varies between 3.5 and 5.0 grams per day. Once in the body, L-arginine is converted into nitric oxide by nitric oxide synthases (NOS), stimulating vessel dilation and improving blood flow to the tissues. Among many things, it has been shown that L-arginine supplementation improves insulin sensitivity and endothelial function in obese patients with type 2 diabetes (77). It is also well known that IR is strongly associated with a reduction in NO availability and endothelial dysfunction. Importantly, ED secondary to reduced NO bioavailability is a very early and prominent feature of IR. It has also been shown that exogenously delivered NO can stimulate insulin transport across endothelial cells and can improve impaired endothelial insulin transport, caused by experimental IR, across endothelial cells (78). Some authors have demonstrated that supplementation with L-arginine, in rats subjected to a high-fat diet, produced a significant improvement in insulin sensitivity, as documented by the reduction in circulating insulin levels and HOMA-IR, and this resulted at least in part related to an increase in adiponectin concentration (79).

In another study, Lucotti et al. demonstrated that treatment with L-arginine, for 21 days, associated with a low-calorie diet and exercise training, in obese patients with type 2 diabetes produced a clear and significant improvement in anthropometric parameters, such as fat mass and waist circumference, and a clear improvement in IR, as documented by the significant reduction in fasting insulinemia and HOMA-IR. The improvement of many other parameters was also documented, including, particularly interesting, the increase in adiponectin and flow mediated dilation, an index of endothelial function (80). So, L-arginine is a very interesting substance, having many benefits for the body and, among these, the action of improving IR should absolutely not be overlooked when we want to implement the prevention of cardiovascular diseases.

## Concluding remarks

As already underlined, despite the considerable progress made in their prevention and treatment, cardiovascular diseases still are the leading cause of death worldwide. IR/Hyperins, despite the extensive scientific literature that demonstrates the deleterious effects on cardiovascular system, has never been taken into consideration, with proper screening and treatment as an independent risk factor for the development of cardiovascular disease. Instead, as well highlighted by wide scientific literature, the effects of a chronically increased insulin levels have proved to be particularly harmful at the level of the cardiovascular system, causing important damages to the heart, brain, kidneys, eyes and peripheral vessels. IR/Hyperins determines the development and progression of atherosclerosis in all vascular districts, and concentric remodeling of the LV which can result in HF and sudden death. For these reasons, we believe that it is extremely important to test for IR/Hyperins subjects suspected of having it, in order to be able to start preventive treatments with the means currently available, as it is done for other recognized cardiovascular risk factors. We are clearly behind schedule.

## Data Availability

The original contributions presented in the study are included in the article/Supplementary Material, further inquiries can be directed to the corresponding author/s.
