# Correction: Insulin resistance/hyperinsulinemia: an important cardiovascular risk factor that has long been underestimated

**DOI:** 10.3389/fcvm.2025.1720220

**Published:** 2025-11-17

**Authors:** Serafino Fazio, Valentina Mercurio, Loredana Tibullo, Valeria Fazio, Flora Affuso

**Affiliations:** 1 Department of Internal Medicine, Federico II University Hospital, Naples, Italy; 2 UOC.Medicina Interna, Azienda Ospedaliera di Caserta, Caserta, Campania, Italy; 3 Independent Researcher, Naples, Campania, Italy

**Keywords:** insulin signaling, insulin resistance, hyperinsulinemia, cardiovascular risk factors, cardiovascular diseases

“cardiovrascular” should be “cardiovascular”.

A correction has been made to the section **Introduction**, paragraph 1.

“It is clear to everyone that, despite the significant progress made in the prevention and treatment of cardiovascular diseases, cardiovascular mortality is still excessively high. It is estimated that every year in Europe there are 2 million deaths from cardiovascular causes (1), and deaths from cardiovascular causes are still in first place among the various causes of death. Indeed, there is a cardiovascular risk factor, which is pathophysiologically connected with the other well-recognized cardiovascular risk factors, treated according to the latest guidelines, which is still not fully taken into consideration: insulin resistance/hyperinsulinemia (IR/Hyperins). This important cardiovascular risk factor is not screened in the general population and, consequently, is not treated, as it is done, instead, with the other recognized cardiovascular risk factors.”

“paucisyntomatic” should be “paucisymptomatic” and “Pub med” should be “PubMed”.

Corrections have been made to the section **Introduction**, paragraph 2.

“It is estimated that IR's prevalence is constantly growing worldwide, reaching up to 51% of the general population, with the highest values for developed and/or developing countries (2). Hyperins is a constant and prominent feature of IR (3, 4). Insulin is a hormone that, in addition to the regulation of glucose metabolism, has important actions in several systems and organs, with the cardiovascular system as one of its main targets, and like all hormones it can cause damage if its levels are outside the normal range (5). Unfortunately, Hyperins that accompanies IR is mostly asymptomatic or paucisymptomatic, making its early diagnosis very difficult. Hence, Hyperins associated with IR lasts, in most cases, for many years before showing itself to have caused some damage or, more often, to have resulted in overt type 2 diabetes (3, 4). In this article we aim at analyzing the evidence in the scientific literature for considering IR/Hyperins as an important CV risk factor, like or more than other well-recognized CV risk factors, and verifying if there are possibilities of mass screening and, consequently, of timely treatment. The scientific literature consulted and used to write the article was found in PubMed, Scopus, Science Direct, etc. using the following keywords: insulin, insulin signaling, insulin resistance, hyperinsulinemia, cardiovascular risk factors, cardiovascular diseases, cardiovascular system. We selected the studies that explored the association between IR/Hyperins and the cardiovascular system, and those that discussed the possibilities of screening and treatment of IR/Hyperins.”

“underdstood” should be “understood” and “micro polycystic ovary syndrome” should be “polycystic ovary syndrome”.

Corrections have been made to the section **Insulin resistance/Hyperinsulinemia**, paragraph 2.

“The causes of IR are various and not entirely understood: overweight and obesity (particularly visceral); sedentary lifestyle; unbalanced diet in favor of excessive carbohydrate intake; chronic stress; prolonged use of diabetogenic drugs; genetic causes; etc. Muscle tissue, adipose tissue, and the liver are the main targets of insulin resistance. The symptoms and signs of IR are scarce and non-specific (drowsiness and tiredness, increased appetite, concentration difficulties, tendency to gain weight, predominantly abdominal adiposity, high levels of LDL cholesterol, high fasting triglyceride levels, tendency to arterial hypertension, etc.) (3, 4, 6). For this reason, IR/Hyperins lasts, often unrecognized, for many years. Diagnosis is easier in subjects with metabolic syndrome and in infertile women with polycystic ovary syndrome, but IR can also be present in subjects that are difficult to suspect, such as in normal weight and thin subjects (7).”

“Carboidrates” should be “carbohydrates” and “Metformine” should be “metformin”.

A correction should be made to the section **Treatment of IR/hyperins**, paragraph 1.

“The first thing to do to improve the condition of IR/Hyperins is a radical change in lifestyle by increasing physical activity and reducing daily caloric intake through a balanced diet not rich in carbohydrates. However, clinical practice shows that only a low percentage of subjects consistently implement these lifestyle changes. For this reason, the majority of the insulin resistant population needs to be helped by adding to their diet substances that increase insulin sensitivity and reduce circulating levels of insulin. There are numerous substances, both drugs and natural substances, that act positively in this sense, but, even today, no substance is authorized for this purpose outside of the diagnosis of diabetes. Among these substances, certainly, Sodium-Glucose cotrasporter2-inhibitors (SGLT2-i), Metformin, Berberine, Glucagon Peptide -1 receptor agonists (GLP-1 Ras) and L-arginine deserve particular attention.”

“Pre-existig” should be “pre-existing”.

A correction has been made to the section **Metformin**, paragraph 2.

“Metformin treatment has also demonstrated notable benefits in patients with HFpEF. In fact, the results of a fairly recent study, carried out using meta-regression analysis on observational and randomized studies, shows that treatment with Metformin significantly reduces (*p* < 0.003) mortality in patients with HFpEF (59). Metformin has been shown to have numerous favorable effects on the heart of subjects with HF. In fact, it improves the energy status of the myocardium as a consequence of the modulation of glucose and lipid metabolism, the reduction of oxidative stress and inflammation, and the reduction of pathological cardiac remodeling (60). A few years ago the results of a meta-analysis were published demonstrating how metformin therapy has favorable effects on left ventricular mass (LVM) and EF both in subjects with and without pre-existing cardiovascular disease (61).”

“fastig insulin” should be “fasting insulin” and “controlled trials” should be “placebo-controlled trials”.

A correction should be made in the section **Berberine**, paragraph 2.

“Berberine, in addition to having many experimental studies to support its positive action on glucose metabolism and IR/Hyperins, also has many clinical studies that support its role in cardiovascular prevention. A review and meta-analysis of randomized controlled trials on berberine has highlighted how it can be a valid alternative, without causing serious adverse reactions, to drugs currently used for cardiovascular prevention (66). In a randomized, placebo -controlled study lasting 4 months, 59 patients with metabolic syndrome were treated with a combination of nutraceuticals, containing berberine 500 mg, or placebo. The results of this study demonstrated that pts treated with berberine had a significant reduction in IR/Hyperins, as documented by reductions in HOMA-IR and fasting insulin levels (39). Furthermore, in these pts, using Doppler-ecocardiography, was documented a significant reduction in LVM and relative wall thickness (RWT), and an improvement in LV diastolic function (40). Another randomized and controlled study of berberine 500 mg per day compared to placebo, carried out in 145 subjects with metabolic syndrome and left ventricular hypertrophy, demonstrated how treatment with berberine for 6 months resulted in a significant reduction (*p* < 0.001) in LVM. For this reason, the authors conclude that this treatment could represent an effective strategy to reduce an important risk factor such as LVM (67).”

“cotrasporter2” should be “cotransporter2”.

A correction has been made to the heading, it now reads “Sodium-glucose cotransporter2-inhibitors”.

“canaglifozin, dapaglifozin and empaglifozin” should be “canagliflozin, dapagliflozin, and empagliflozin”.

A correction has been made to the section **Sodium-glucose cotransporter2-inhibitors**, paragraph 1.

“Sodium-glucose cotransporter2 inhibitors (SGLT2-i) are a relatively recent class of oral drugs, approved for the treatment of adults with type 2 diabetes and, recently, also for the treatment of HF (50, 51). They include canagliflozin, dapagliflozin, and empagliflozin. A vast scientific literature is now available demonstrating how treatment with SGLT2-i reduces the risk of hospitalization, death from cardiovascular events and all causes of death in patients with HF (52). A recent study on 6,263 patients >40 years old with EF > 40%, assigned to receive dapagliflozin (10 mg once daily) or placebo in addition to standard therapy, demonstrated a significant reduction in the risk of worsening of HF and death from cardiovascular events (53). Another recent study of metaregression-analysis of vaste proportion, aimed at assessing the effects of these drugs on all-cause mortality, has shown that SGLT2-i reduce all-cause mortality in randomized trials (54).”

“GLP-1 Ras” should be “GLP-1 RAs”, “delaglutide” should be “dulaglutide”, “lixiglutide” should be “lixisenatide”

Corrections have been made to the section **Glucagon-like peptide 1 receptor agonists**, paragraphs 1, 2 and 3.

“Glucagon-like peptide 1 receptor agonists (GLP-1 RAs) are a class of drugs not long ago authorized for the treatment of type 2 diabetes and obesity. They are administered by subcutaneous injection and are quite expensive drugs. This class of drugs includes semaglutide, albiglutide, dulaglutide, exenatide, liraglutide, and lixisenatide. It seems that they act not only by reducing body weight, but also by acting on mechanisms involved in the determinism of IR/Hyperins, such as, for example, increasing the expression of glucose transporters in insulin-dependent tissues, reducing inflammation and oxidative stress, and modulating lipid metabolism (69, 70).”

However, although GLP-1 RAs have actually been shown to improve cardiovascular risk factors such as body weight, blood pressure, LDL cholesterol and triglycerides, and glycemic control, they have been shown to reduce all-cause mortality in patients with type 2 diabetes at high risk of cardiovascular events, but have not reduced cardiovascular mortality, non-fatal myocardial infarction, non-fatal stroke and hospitalizations for HF (71, 72). Another recent meta-analysis carried out on 54,092 pts from 7 randomized placebo-controlled trials on the use of GLP-1 RAs in subjects with type 2 diabetes, of whom 16% also had a history of HF, demonstrated that these drugs appear to protect the diabetic population from the development of HF, but, in subjects with pre-existing HF, they do not reduce the onset of episodes of HF exacerbation with consequent hospitalization, nor mortality (73). A further recent meta-analysis carried out to verify whether treatment with GLP-1 RAs in subjects with HF, with or without type 2 diabetes, could lead to a reduction in morbidity and mortality compared to placebo, also demonstrated that this therapy did not reduce the number of major adverse cardiovascular events, including cardiovascular mortality or reduction in hospital admissions for HF, and did not lead to an improvement in HF or six-minute walking test (74).

In addition, recently, numerous reports of serious adverse events have been published, such as cases of severe pancreatitis. In fact, patients taking either semaglutide or liraglutide had nine times an elevated risk for pancreatitis but, also, they had a very high risk to develop bowel obstruction, and to experience gastroparesis (75). Furthermore, European Medicine Agency published a statement on ongoing review of GLP-1 RAs about the possibility that liraglutide and semaglutide can stimulate suicidal thoughts and self-injury, made by the Icelandic medicines agency (76).”

“propre screening” should be “proper screening”.

A correction should be made in the section **Concluding remarks**.

“As already underlined, despite the considerable progress made in their prevention and treatment, cardiovascular diseases still are the leading cause of death worldwide. IR/Hyperins, despite the extensive scientific literature that demonstrates the deleterious effects on cardiovascular system, has never been taken into consideration, with proper screening and treatment as an independent risk factor for the development of cardiovascular disease. Instead, as well highlighted by wide scientific literature, the effects of a chronically increased insulin levels have proved to be particularly harmful at the level of the cardiovascular system, causing important damages to the heart, brain, kidneys, eyes and peripheral vessels. IR/Hyperins determines the development and progression of atherosclerosis in all vascular districts, and concentric remodeling of the LV which can result in HF and sudden death. For these reasons, we believe that it is extremely important to test for IR/Hyperins subjects suspected of having it, in order to be able to start preventive treatments with the means currently available, as it is done for other recognized cardiovascular risk factors. We are clearly behind schedule.”

The reference for 33 was erroneously written as “Hoang K, Zhoo Y, Gardin JM, Carnethon M, Makunal K, Yanez D, et al. Left ventricular mass ad predictor of cardiovascular disease events in older adults with and without metabolic syndrome and diabetes. *JACC Crdiovasc Imaging*. (2015) 8(9):1007–15. doi: 10.1016/j.jcmg.2015.04.019”. It should be “Hoang K, Zhoo Y, Gardin JM, Carnethon M, Makunal K, Yanez D, et al. Left ventricular mass as predictor of cardiovascular disease events in older adults with and without metabolic syndrome and diabetes. *JACC Cardiovasc Imaging*. (2015) 8(9):1007–15. doi: 10.1016/j.jcmg.2015.04.019”.

The reference for 58 was erroneously written as “Campbell JM, Bellman SM, Stephenson MD, Lisy K. Metformin reduces all-cause mortality and disorders of aging independent of ita effect on diabetes control. A systematic review. *Aging Res Rev*. (2017) 40:31–44. doi: 10.1016/j.arr.2017.08.003”. It should be “Campbell JM, Bellman SM, Stephenson MD, Lisy K. Metformin reduces all-cause mortality and disorders of aging independent of its effect on diabetes control. A systematic review. *Aging Res Rev*. (2017) 40:31–44. doi: 10.1016/j.arr.2017.08.003”.

The reference for 69 was erroneously written as “Bednarz K, Kowalczyk K, Cwynar M, Czapla D, Czarkowski W, Imita D, et al. The role of GLP-1 receptor agonists in insulin resistance with concomitant obesity treatment in polycystic ovary syndrome. *Int J Miol Sci*. (2022) 23(8):4334. doi: 10.103390/ijms23084334”. It should be “Bednarz K, Kowalczyk K, Cwynar M, Czapla D, Czarkowski W, Imita D, et al. The role of GLP-1 receptor agonists in insulin resistance with concomitant obesity treatment in polycystic ovary syndrome. *Int J Mol Sci*. (2022) 23(8):4334. doi: 10.103390/ijms23084334”.

The reference for 79 was erroneously written as “Miczke A, Sulisburska J, Pupek-Musialik D, Ostrowska A, Jablecka A, Krejpcio Z, et al. Effect of L-arginine supplementation on insulin resistance and serum adiponectin concentration in rats with far diet. *Int J Clin Exp Med*. (2015) 8(7):10358–66. PIMD: 26379826”. It should be “Miczke A, Sulisburska J, Pupek-Musialik D, Ostrowska A, Jablecka A, Krejpcio Z, et al. Effect of L-arginine supplementation on insulin resistance and serum adiponectin concentration in rats with fat diet. *Int J Clin Exp Med*. (2015) 8(7):10358–66. PIMD: 26379826”.

The original version of this article has been updated.

